# Consolidation in the dental industry: a closer look at dental payers and providers

**DOI:** 10.1007/s10754-019-09274-x

**Published:** 2019-10-03

**Authors:** Kamyar Nasseh, John R. Bowblis, Marko Vujicic, Sean Shenghsiu Huang

**Affiliations:** 1 Health Policy Institute, American Dental Association, 211 E. Chicago Ave., Chicago, IL 60611-2637 USA; 2 Department of Economics, Miami University, Oxford, OH 45056 USA; 3 Department of Health Systems Administration, Georgetown University, Washington, DC 20057 USA

**Keywords:** Dental insurance, Market structure, Dental care, Consolidation, I11, I13, L40, L44

## Abstract

We examine the effect of commercial dental insurance concentration on the size of dental practices, the decision of dentists to own a practice, and the choice of dentists to work at a dental management service organization—a type of corporate group practice that has become more prevalent in the United States in recent years. Using 2013–2015 dentist-level data from the American Dental Association, county-level data on firms and employment from the United States Census, and commercial dental insurance market concentration data from FAIR Health^®^, we find a modest effect of dental insurance market concentration on the size of dental practices. We also find that a higher level of commercial dental insurance market concentration is associated with a dentist’s decision not to own a practice. There is inconclusive evidence that higher levels of dental insurance market concentration impact a dentist’s decision to affiliate with a dental management service organization. Overall, our findings imply that dentists consolidate in response to increases in concentration among commercial dental insurers.

## Introduction

**Fig. 1:**
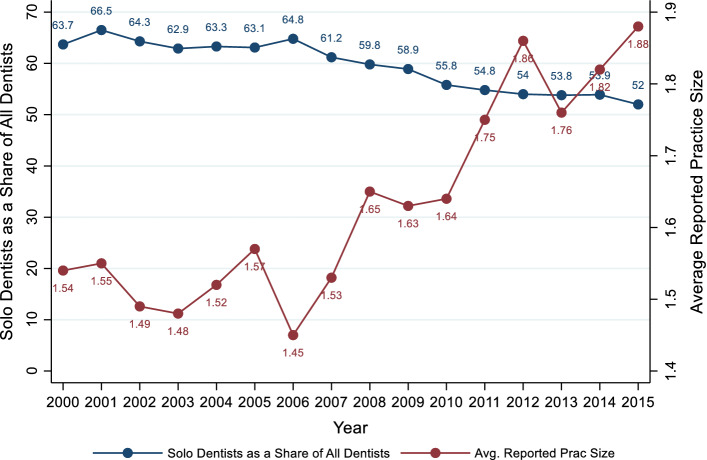
Solo dentists and reported practice size. *Notes*: A solo dentist works in a practice with no other dentist. Distribution of Dentists results based on 3 pooled years of data per data point. *Source*: Data on the share of solo practice dentists are from the 2000–2009 American Dental Association Survey of Dental Practice and the 2010–2015 American Dental Association Distribution of Dentist Survey. Data on reported practice size are from the 2000–2015 American Dental Association Survey of Dental Practice

Traditionally, U.S. dental practices are small operations, consisting of no more than a few dentists that have ownership interests in the business. Over time, more dentists have become affiliated with larger organizations, no longer own their practices, and have become salaried employees. (Guay and Wall 2016; Nasseh and Vujicic 2018; American Dental Association 2018). As shown in Fig. 1, between 2000 and 2015, the average reported practice size increased and the percentage of dentists in solo practice declined from 64 to 52%. While historical data on large dentist chains, called dental management service organizations (DMSOs) is lacking, there is some evidence DMSOs are playing a bigger role in dentistry (Guay et al. 2014; American Dental Association 2017a; Nasseh and Vujicic 2017). These organizations are entities where marketing, business and administrative responsibilities are handled centrally (Winegarden and Arduin 2012). Large organizations like DMSOs also have the ability to negotiate the prices of dental services on behalf of their dentists (Kuttler 2017).

There are multiple reasons why dentists may be organizing into larger practices. The first reason is work-life balance. A solo practice dentist is almost entirely responsible for paying their employees and for the success of the business, leading to significant stress for some owners. In a larger practice, these responsibilities are handled by a professional office. Second, the cost of going to dental school and setting up a practice has been increasing (Asch et al. 2013). New dentists may prefer to start off as salaried employees or work at a larger practice for greater financial stability. The third reason, and the focus of this paper, is the relative bargaining position between dentists and dental insurers.

A dentist’s income is tied to the number of patients treated and the reimbursement rate received for those treatments. However, higher reimbursement rates may pressure dental insurers to charge higher insurance premiums in order to maintain the same level of profitability. If employers, who pay for most of the commercially insured dental insurance plans (National Association of Dental Plans 2017) find these premiums to be too high, they could switch to another dental insurer or drop dental coverage altogether. This means that dental insurers have an incentive to put downward pressure on reimbursement rates whereas dentists aim to receive more compensation for their services. The net effect on provider prices depends on the relative bargaining strength of providers and insurers (Trish and Herring 2015). In medical markets, concentrated insurance markets are also associated with larger physician practices and a greater likelihood that physicians are directly employed by hospitals (Brunt and Bowblis 2014; McCarthy and Huang 2018). As a result, consolidation of providers has counteracted potentially lower prices that can arise from health insurance consolidation (Gaynor and Town 2012; Dafny et al. 2018). The net result is higher prices for medical services (Austin and Baker 2015; Dunn and Shapiro 2014).

We explore the relationship between dental insurer concentration and the organizational structure of dental practices. To the best of our knowledge, we are one of, if not the first, to study the impact of insurer concentration on the consolidation of dental practices. Understanding this relationship in dental markets is important because in 2016, dental markets represented $124 billion in health care spending (Garvin 2017). The organizational structure of dental practices has been changing since 2000 (Fig. 1). Data from the American Dental Association (ADA) found that the percentage of dentists who are practice owners is at an all-time low (American Dental Association 2018) and that dental group practices in which dentists are part of a regional or national organization are more prevalent (Guay et al. 2014). Our paper provides important empirical evidence of the dynamics between insurer concentration and provider consolidation in U.S. dental markets.

## Conceptual framework

Medical markets have experienced significant insurer and provider consolidation (United States Government Accountability Office 2009; Dafny 2015; Dafny et al. 2012). Dental and medical insurance are similar in many ways, particularly the fact that most medical and dental insurance is provided through employer-sponsored plans (National Association of Dental Plans 2017). Yet, consumers are more sensitive to changes in the prices of dental services and dental insurance has higher levels of cost sharing (American Dental Association 2017b). Moreover, typical dental treatments are lower in cost relative to treatments covered by medical insurance, such as those for heart attacks. Even in some high-cost cases, the maximum dollar amount of services covered by dental insurance is limited each year, lessening the need for insurers to contain the high cost of some patients. While differences exist between medical and dental insurance that may impact the relative bargaining position between providers and insurers in both markets, the fundamentals of how providers and insurers bargain are the same.

Commercial insurers engage in bilateral Nash bargaining with providers in the medical care markets (Dunn and Shapiro 2014; Ho and Lee 2017). Whether provider prices are higher or lower in a given market will depend on the relative bargaining leverage of each party. Insurers that capture a greater share of enrollees in a market are able to exert more bargaining leverage vis-à-vis providers by credibly excluding providers from their networks. As a result, monopolistic insurers in competitive provider markets can induce providers to bid down the prices of procedures toward marginal cost. There is evidence of this phenomenon in dental markets. For the period of 2011 to 2015, Figs. 2a and b use 3-digit zip code data from the FAIR Health^®^ Dental Module to report the percentage change in list prices for dental services against the percentage change in dental insurer market concentration, as measured by the Herfindahl–Hirschman Index (HHI). For both adult prophylaxes (i.e. teeth cleaning) and a price index of eight common dental procedures, markets which became more concentrated show the lowest percentage increase in list prices. Therefore, to counteract the bargaining leverage of insurers, providers can form group practices or health systems. By forming larger organizations, providers become “must-have” providers and can credibly exclude an insurer’s enrollees from their services. As providers gain negotiating leverage, they may be able to bid the price of their services toward the monopolistic profit-maximizing price. However, this consolidation among dentists may need to meet a certain threshold before it can counteract the bargaining leverage of insurers.

**Fig. 2:**
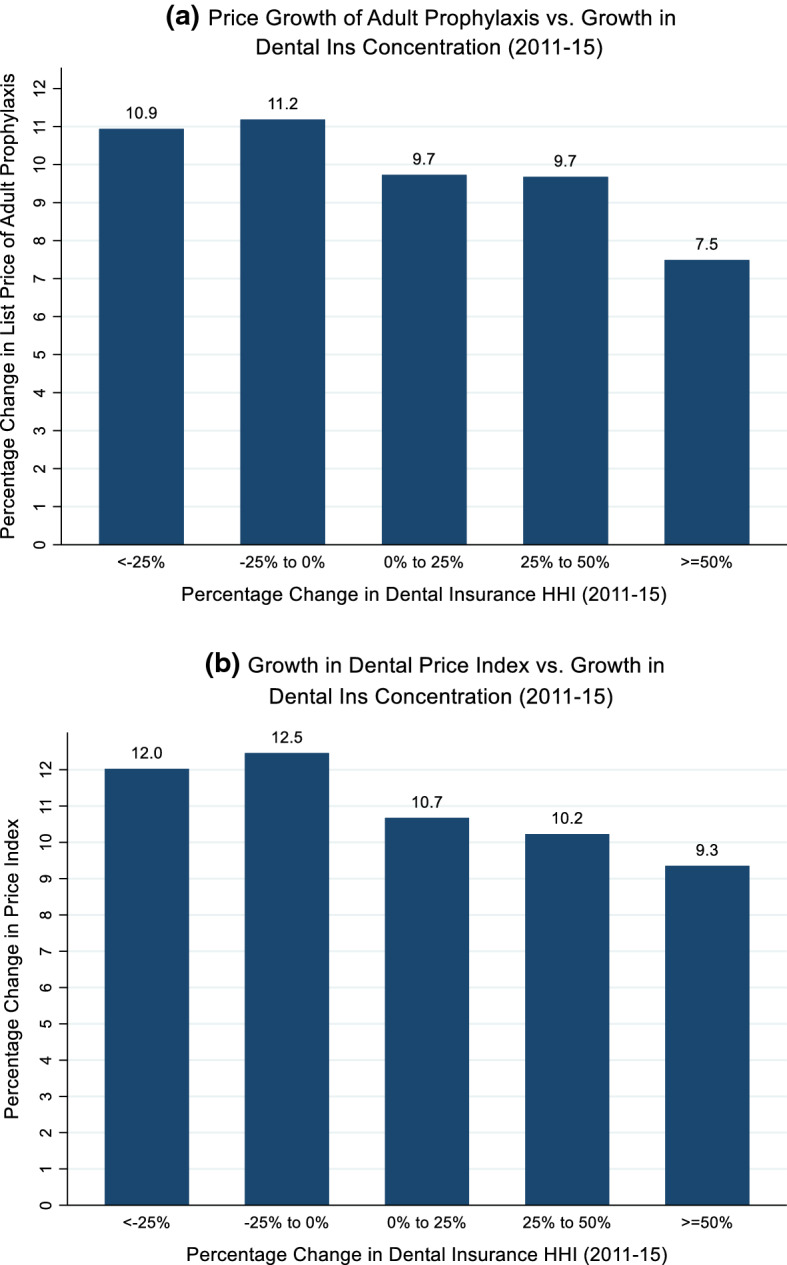
Relationship between Percentage Change in Commercial Dental List Prices and Percentage Change Dental Insurance Concentration (2011–2015). *Notes:* D1110-Adult Prophylaxis. Price index based on eight common dental procedures (D0120, D0150, D0220, D0230, D0274, D1110, D1120, D2392). Dental price index calculated using weights generated from the 2015 FAIR Health^®^ Dental Module based on total billings. *Source*: Analysis performed using 3-digit zip code data from the 2011 and 2015 FAIR Health^®^ Dental Module

Within this framework, the party with a weaker bargaining position often strategizes to enhance its bargaining position. These dynamics have been playing out ever since commercial health insurers introduced managed care, a type of health insurance that steers patients to lower cost hospitals and physicians in exchange for lower insurance premiums (Wu 2009; Morrisey 2001). It remains to be seen whether a similar dynamic is playing out between dental providers and insurers.

**Fig. 3:**
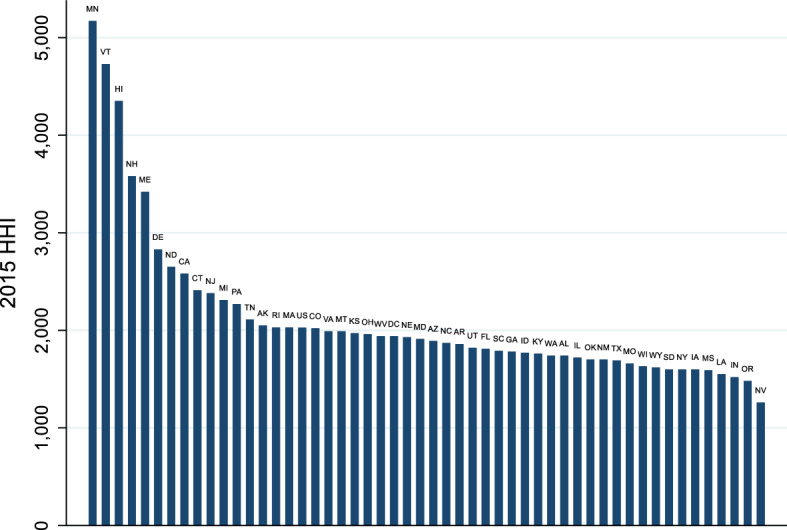
State level commercial dental insurance concentration *Source* 2015 FAIR Health^®^ dental module

Dental insurance markets are moderately concentrated. Nationally, from 2011 to 2015, the HHI among dental insurers nationally remained relatively flat, between 2000 and 2200. However, there is significant variation in dentist insurer concentration across states (Fig. 3), and some states have experienced significant changes in insurer concentration.

While the national trend toward consolidation among dental practices could be explained by a number of factors, bargaining dynamics can be different on a state-by-state basis, and may become increasingly important as dental practices become larger. Therefore, it is our hypothesis that the organizational structure of dental practices is partially a response to changes in dental insurance market concentration, similar to how physicians merged into larger practices or affiliated with hospitals in markets where health insurer concentration was higher in order to strengthen their bargaining position (Brunt and Bowblis 2014; McCarty and Huang 2018). We also expect to find that in markets with greater concentration among dental insurers, dentists are more likely to work for larger practices, not have ownership interests in those practices, and are more likely to be employed by DMSOs.

## Data and methods

### Data sources and sample

To construct an analytic sample of actively practicing dentists in the U.S., we used a number of data sources, including those collected and maintained by the American Dental Association (ADA): the ADA Distribution of Dentists (DOD) Survey and the ADA office database. These databases include information on individual dentists (e.g., dentist demographics, specialty information, employment relationship) and the organizational structure of the practices in which they work (e.g., practice size, DMSO affiliation). Since the DOD is collected from approximately one-third of U.S. dentists each year, we used data from the three most recent years available (2013, 2014 and 2015) to reflect the status of all dental practices. Based on the ADA’s masterfile of all actively practicing dentists, the 2013–2015 DOD has about a 70.6% response rate. The ADA data was merged with measures of dental insurance market concentration obtained from the FAIR Health^®^ Dental Module based on the year the dentist was surveyed. The FAIR Health^®^ Dental Module contains dental claims for approximately 75% of individuals who have commercial dental insurance in the United States (FAIR Health^®^ Inc. 2016). Finally, the FAIR Health^®^ Dental Module and ADA databases were supplemented with publicly available information from the Area Health Resource File (AHRF) and the U.S. Census Statistics of U.S. Businesses (United States Census 2018).

Using this data, the analytic sample was restricted to dentists that were actively practicing in the United States and reported information about the organizational structure of their practice. This resulted in a sample of 53,831 dentists. After we restricted the sample to urban dentists and those with non-missing data, the final analytic sample for the main analysis was 46,594 unique respondent dentists. We restricted our sample to urban dentists since rural geographic areas are typically larger in size and may not accurately represent a rational dental insurance market. While data from 2013 through 2015 was utilized, this analytic sample was cross-sectional in nature and approximated the state of the dental industry around 2015.

### Dental insurance market concentration

In a national study, it is not feasible to perform a detailed analysis to define each geographic market for dental insurance; therefore, proxies are utilized. Some studies have used the state as a proxy (Moriya et al. 2010), arguing that insurers are regulated at the state level and that once entry occurs, it is easy to expand into other locations within the state. However, this may result in geographic markets that are significantly large, underestimating the level of dental insurer concentration. Therefore, we referred to previous work that defines insurance markets based on the first three digits of a zip code (Dafny et al. 2012). While market concentration has been calculated using multiple approaches (Austin and Baker 2015; Baker et al. 2014), we did not have access to the detailed claims data from FAIR Health^®^. Therefore, we were restricted to calculating insurer concentration based on a fixed geographic area, which, based on the level of granularity provided by FAIR Health^®^, is a 3-digit zip code.

Using the 3-digit zip code to proxy for the geographic market, FAIR Health^®^ provided dental insurer market concentration as measured by the Herfindahl–Hirschman Index (HHI) for each year from 2011 through 2015. The calculation of HHI is based on the market share of each insurers’ number of paid dental claims. Some prior studies have used enrollment data to calculate insurer HHIs. We used paid claims to calculate market share because dentists may not have enrollment information but knew how many claims were submitted to various dental insurers. Therefore, an HHI based on paid claims may better reflect the insurance market dynamics that dentists face when determining how to organize their practice.

### Organizational structure of dental practice

We examined three dependent variables that measured the organizational structure of the dental practice of the respondent. The first dependent variable was the practice size where the respondent dentist works. Larger practice sizes can theoretically increase the bargaining leverage of dentists against commercial insurers, and we measured practice size as the number of dentists in the practice. We eliminated outliers by excluding dentists that reported extremely large practice sizes (the top 0.02%, which is 141 + dentists). The second dependent variable was whether the respondent dentist has an ownership interest in the dental practice. Because most dentists have an equity interest in their practice, we operationalized this dependent variable as a binary variable equal to 1 if the dentist reports he is a non-owner and equal to 0 if the dentist reports having an ownership interest in the practice. The third dependent variable was whether the dentist is part of a dental management service organization (DMSO).

### Empirical strategy

Our analysis evaluated the impact of dental insurance market concentration on the three measures of organizational structure of dental practices. To understand this relationship, we evaluated the following model:

1 $$ Y_{i} = {\text{G}}(Insurance \,Concentration_{i} *\beta + {\mathbf{X}}_{i}\varvec{\alpha}\text{)} $$

where *i* indexes a dentist, $Y_{i}$ is a measure of the organizational structure of the practice, dental insurance concentration is measured by a HHI in the 3-digit zip code of the dentist, and ${\mathbf{X}}_{i}$ is a set of control variables. We used HHI in its logged and level form in Eq. 1. We reported both results and confirmed that they are quantitatively similar. Because all of the practice structure measures we examined are non-linear, we needed to use the proper functional form of $G\left( - \right)$ to capture the non-linear nature of each dependent variable. We used Poisson regression when analyzing the number of dentists in a practice and estimated probit models when determining the likelihood that a dentist is a non-owner or part of a DMSO. Standard errors were clustered at the 3-digit zip code level.

According to the conceptual model, we expected that more concentrated dental insurance markets will lead to consolidation among dental providers, which can be measured as larger practices, a lower percentage of dentists with ownership stakes, and a greater share of dentists in DMSOs. In all cases, this implies that we expected the marginal effect associated with $\beta$ to be positive. To account for differences in dentists and where they practice, $\varvec{X}_{\varvec{i}}$ is a set of control variables that are commonly adopted in the literature. These include the dentist’s experience (measured as years), experience squared, gender, race/ethnicity, and dental specialty (i.e., general practice dentist, pediatric dentist, and other specialists). We also controlled for county characteristics, including census region, population density (population per square mile), dentist density (professionally active dentists per square mile), an indicator for whether the local market is a dental health professional shortage area (HPSA), log of real median household income, log of total population, and a metro continuum categorical variable. We also included year indicators.

While a number of studies in the medical literature estimate Eq. (1) treating the relationship between concentration in health insurance and medical provider markets as exogenous (Trish and Herring 2015; Moriya et al. 2010), it has been argued that health insurer HHI is endogenous. This could be due to measurement error (Dafny et al. 2011) in calculating insurer HHI or potential for reverse causality where insurers merge or exit the market in response to the consolidation of providers (Brunt and Bowblis 2014; McCarthy and Huang 2018; Dunn and Shapiro 2014). Therefore, we also estimated Eq. (1) under the assumption that dental insurer HHI is endogenous. Specifically, we estimated a first-stage model as follows:

2 $$ Insurance\, Concentration_{i} = \varvec{Z}_{\varvec{i}}\varvec{\delta}+ {\mathbf{X}}_{\varvec{i}}\varvec{\gamma}+ v_{i} $$

where ${\mathbf{Z}}_{\varvec{i}}$ are excluded instruments and $v_{i}$ is an error term. We then applied a control function approach (Wooldridge 2015), commonly referred to as two-staged residual inclusion (2SRI) in which the estimated residual in Eq. (2) is used as a regressor in Eq. (1). This 2SRI approach addresses the potential for endogeneity of dental insurer HHI and will produce consistent estimates of $\beta$ in Eq. (1) when there exists at least one excluded instrument that predicts insurer HHI but does not directly affect the organizational structure of dental practices.

Following the medical insurance literature, there were two sets of instrument variables that we used. Both attempted to measure the level of concentration among insurers by measuring whether a market is likely to face entry or exit by an insurer and the ability of an insurer to maintain or increase market share.

The first set of instrumental variables used in the medical literature is the number of firms and number of employees per firm (e.g. firm size), both measured at the county level (Brunt and Bowblis 2014; Bates and Santerre 2008; Town et al. 2007; Dranove et al. 1998; Baker and Brown 1999). These studies argue that because most individuals have insurance coverage through their employer, as is the case in dental markets (National Association of Dental Plans 2017), an insurance market’s attractiveness and an insurer’s market share will depend on the size and distribution of employers. That is, the number of firms approximates the size and profitability of local commercial dental insurance markets. As such, a larger number of firms is found to be negatively correlated with the insurance HHI. On the other hand, firm size is found to be positively correlated with insurance HHI because larger employers may negotiate lower premiums and hence reduce insurers’ incentives toward entry. These market dynamics also apply to the dental industry because the majority of Americans receive dental benefits from commercial entities (American Dental Association 2017c), and 92% of commercial dental plans are financed through group or employer coverage (National Association of Dental Plans 2017). Furthermore, firm size and the number of firms are not likely correlated with the organizational structure of dental practices because dentists provide care to patients from different firms. This implies that the long-term organizational decisions made by dentists are based on the potential actions of commercial dental insurers and the size of the total market, not the decision of any particular employer in the market.

The second set of excluded instruments included the unemployment rate and whether the county has a high proportion of elderly residents, defined as being among the top 5% in the nation with the highest percentage of individuals aged 65 and older (Berry and Waldfogel 2001; Davis 2006). These instruments were found to be valid in studies of physician markets because health insurance is primarily purchased through employers, and markets with stronger economies and larger working age populations are more attractive for health insurers (Dunn and Shapiro 2014). Similar to health insurance, most dental insurance is also purchased through employers, with insurance coverage rates for private dental benefits at 58.1% for working-age adults but only at 27.9% for individuals aged 65 and older (Nasseh and Vujicic 2016). This implies that dental insurers are more likely to enter markets with strong economies (e.g., lower unemployment rates). For older individuals, most have dental coverage through Medicare Advantage. If there are a large number of seniors in an area, we expected dental markets to be less concentrated as various Medicare Advantage plans may team with different dental insurers or offer their own dental insurance in order to attract seniors to their Medicare Advantage plans.

While higher unemployed and elderly populations predict dental insurer HHI, they do not necessarily affect the organizational structure of dental practices. To establish a dental practice, dentists are faced with many fixed costs, including but not limited to finding a location, investing in specialized equipment, and customizing an office to meet their needs. Most importantly, they must undergo the sunk cost of establishing a reputation and patient panel. Due to the high costs associated with establishing a dental practice, it is less likely that established dental practices will change their organizational structure in the short run in response to local economic conditions. It is possible that local economic conditions could have greater influence on the career choices of dentists who just graduated from dental school, but these individuals make up a small proportion of dentists in any market. Therefore, these instruments are likely to satisfy the criteria that they do not influence the organizational structure of dental practices. To assure our results were not sensitive to new dentists in the sample, we also ran a robustness check that is limited to dentists with at least 5 years of experience.

## Results

Table 1 reports summary statistics. The average dentist was in a practice with 2.08 dentists and was an owner in the practice (13.6% are non-owners). Only 3.5% of dentists worked in a practice that was part of DMSO. The average dentist worked in a geographic area with a moderately concentrated dental insurance market (HHI = 2303) (United States Department of Justice 2018), though there was significant variation in the level of insurer concentration. While the data is not shown, 21.8% of dentists worked in highly concentrated dental insurance markets (HHI > 2500), compared to only 3.3% of dentists that worked in unconcentrated dental insurer markets (HHI < 1500). Approximately 79% of respondents were general practitioners, with the remaining dentists being pediatric dentists (4%) or other specialists (17%). The average age of respondents was 53.3 years and the average length of experience for dentists in our sample was 25.8 years. About 76.4% of responding dentists were male, 80.4% were white, and 12.9% practiced in a dental HPSA.

**Table 1:** Summary Statistics (N = 46,594). *Sources*: 2013–2015 American Dental Association Distribution of Dentist Survey, 2015 American Dental Association Dentist Office Database, 2013–2015 FAIR Health^®^ Dental Module, Area Resource File, 2013–2015 U.S. Census Statistics of U.S. Businesses

Variable	Mean	(SD)
*Dependent variables*		
Total dentists in practice	2.075	(3.635)
Non-owner dentist	0.136	(0.343)
DMSO dentist	0.035	(0.184)
*Key variable of interest and instruments*		
3-digit zip code insurer HHI	2303.332	(757.358)
3-digit zip code log of insurer HHI	7.701	(0.272)
Total county firms	29,116.500	(45,277.860)
County employees per firm	19.145	(4.578)
Log of total firms	9.482	(1.292)
Log of employees per firm	2.922	(0.251)
County in top 5% in terms of percentage of population aged 65 and older	0.011	(0.103)
Unemployment rate	6.170	(1.770)
*Dentist specialty*		
General practice dentist	0.785	(0.411)
Pediatric dentist	0.043	(0.203)
Other dental specialty	0.172	(0.377)
*Dentist demographics*		
Experience (years)	25.811	(12.454)
Age	53.290	(11.960)
Male	0.764	(0.425)
White	0.804	(0.397)
Black	0.021	(0.143)
Hispanic	0.036	(0.187)
Asian	0.107	(0.309)
Other race	0.025	(0.157)
Race missing	0.007	(0.080)
*Regional and market controls*		
Northeast	0.204	(0.403)
Midwest	0.222	(0.415)
South	0.301	(0.459)
West	0.273	(0.446)
Population per square mile	1902.464	(5331.515)
Dentist per square mile	1.917	(8.422)
Log real median household income	10.994	(0.242)
Log of county population	13.346	(1.244)
Metro over 1 million	0.649	(0.477)
Metro 250,000 to 1 million	0.245	(0.430)
Metro less than 250,000	0.105	(0.307)
Dental HPSA	0.129	(0.336)
*Year of survey response*		
Year 2013	0.353	(0.478)
Year 2014	0.356	(0.479)
Year 2015	0.292	(0.455)

*HHI* Herfindahl–Hirschman index, *DMSO* dental management service organization. Standard deviation in parentheses

To understand the effect of dental insurer HHI on the organizational structure of dental practices, we started by estimating Eq. (1) under the assumption that insurer HHI is exogenous. These results are reported in Table 2. In Panel A, which measures HHI in logged form, we report the coefficient estimates and average partial effects (APEs) for a 10% increase in dental insurer HHI. A 10% increase in dental insurer HHI corresponded to an approximate 230 point increase in the HHI for the average dentist in the sample. In Panel B, which measures HHI in level form, we report coefficient estimates and APEs corresponding to a 1000 point increase in HHI.

**Table 2:** Relationship between dental insurance market concentration and dental practice characteristics. *Source*: 2013–2015 American Dental Association Distribution of Dentist Survey, 2015 American Dental Association Dentist Office Database, 2013–2015 FAIR Health^®^ Dental Module, Area Health Resource File

Dependent variable	Poisson	Probit	Probit
	(1)	(2)	(3)
	Total dentists in primary practice	Non-owner	DMSO dentist
*Panel A*			
log(HHI)	0.298**	0.134***	− 0.026
	(0.132)	(0.050)	(0.075)
APE of log(HHI)	0.619**	0.025***	− 0.002
	(0.279)	(0.009)	(0.005)
*Panel B*			
HHI in 000s	0.121***	0.054***	0.009
	(0.046)	(0.018)	(0.025)
APE of HHI in 000s	0.251**	0.010***	0.0007
	(0.097)	(0.003)	(0.002)
Number of observations	46,594		

Regressions include year effects, gender, experience, experience squared, county dental HPSA designation, log of real median household income, population density, dentist density, census regions, urban continuum controls, dentist race/ethnicity, log of county population, and dentist specialty (GP, pediatric, other specialty). Dental insurance market concentration measured by the log or level of insurer HHI at 3-digit zip code level. Standard errors clustered at the 3-digit zip code level. ***p < 0.01, **< 0.05,*p < 0.10

The logged HHI specification finds a 10% increase in dental insurer HHI was associated with 0.06 dentist increase in practice size. A similar increase in dental insurer HHI was associated with a 0.25 percentage point increase in the probability that a dentist was a non-owner. Using the level HHI specification, a 1000 point increase in HHI was associated with a 0.25 dentist increase in practice size and a 1 percentage point increase in the probability that a dentist was a non-owner. There was not a statistically significant association between concentrated dental insurance markets and the likelihood that a dentist was part of a DMSO in either specification. This implies that the models that specify dental insurer HHI in logged or level form come to quantitatively similar conclusions. Specifically, we find that more concentrated dental insurance markets were associated with larger dental practices and lower rates of ownership, but did not drive dentists to be part of DMSOs.

The results for when dental insurer HHI was treated endogenously are reported in Table 3. Columns (1), (3) and (5) in the top panel of the table report the APEs for a 10% increase in dental insurer HHI using a logged HHI specification. Columns (2), (4) and (6) report the APEs for a 1000 point increase in dental insurer HHI using a level HHI specification. The bottom panel reports the corresponding first stage results (Eq. 2). Columns (1) and (2) treat insurer HHI as exogenous with the remaining columns treating insurer HHI as endogenous. In almost all cases, the first stage F-statistics on the excluded instruments are over or very close to 10, which is consistent with the threshold suggested for weak instrument tests (Staiger and Stock 1997). Moreover, the first stage residuals suggested that insurer HHI is endogenous in the non-ownership model (Columns (3) and (4)) when log of firm size and log of number of firms are used as instruments (*p* value < 0.05), but for all other specifications and dependent variables, the first stage residuals were not found to be statistically significant (not reported for brevity). This indicated that insurer HHI is likely not endogenous.

**Table 3:** Average partial effects of dental insurer HHI on practice size, non-ownership and DMSO status. *Source*: 2013–2015 American Dental Association Distribution of Dentist Survey, 2015 American Dental Association Dentist Office Database, 2013–2015 FAIR Health^®^ Dental Module, Area Health Resource File, 2013–2015 U.S. Census Statistics of U.S. Businesses

Outcome variables	Exogenous dental insurer log(HHI)	Exogenous dental insurer level HHI	Endogenous dental insurer log(HHI)	Endogenous dental insurer level HHI	Endogenous dental insurer log(HHI)	Endogenous dental insurer level HHI
	(1)	(2)	(3)	(4)	(5)	(6)
Total dentists in primary practice	0.619**	0.251**	0.701	0.302*	0.839**	0.338**
	(0.279)	(0.097)	(0.451)	(0.170)	(0.368)	(0.141)
Non-owner	0.025***	0.010***	0.100**	0.037**	0.043	0.016
	(0.009)	(0.003)	(0.046)	(0.018)	(0.031)	(0.011)
DMSO dentist	− 0.002	0.0007	0.031	0.012	− 0.017	− 0.004
	(0.005)	(0.002)	(0.022)	(0.008)	(0.026)	(0.009)
*First stage excluded instruments*						
log(Employees per firm)			0.090**	0.275**		
			(0.044)	(0.119)		
log(Total firms)			− 0.239***	− 0.607***		
			(0.062)	(0.179)		
Unemployment rate					− 0.206***	− 0.632***
					(0.062)	(0.190)
(Unemployment rate)^2^					0.023***	0.067***
					(0.006)	(0.017)
(Unemployment rate)^3^					− 0.0006***	− 0.002***
					(0.0001)	(0.0004)
County in Top 5% in terms of population 65 and older					− 0.123***	− 0.242***
					(0.031)	(0.075)
F-test for weak IVs	N/A	N/A	11.04	9.88	12.20	11.18
(*P*-value) F-test for weak IVs			0.0000	0.0001	0.0000	0.0000

Number of Observations—46,594. Regressions include year effects, gender, experience, experience squared, county dental HPSA designation, log of real median household income, population density, dentist density, census regions, urban continuum controls, dentist race/ethnicity, log of county population, and dentist specialty (GP, pediatric, other specialty). Dental insurance market concentration measured by level (in 000s) or log of insurer HHI at 3-digit zip code level. We use generalized method of moments to generate standard errors clustered at the 3-digit zip code level when estimating a Poisson model. In the probit models, if the coefficient on residual term generated from the first stage is statistically significant at the 5% level, we estimate clustered bootstrapped standard errors with 1000 replications in order to perform proper inference on β. These standard errors are also clustered at the 3-digit zip code level. ***p < 0.01, **p < 0.05, *p < 0.10

Compared to the exogenous results, when we treated insurer HHI as endogenous, we came to the same general conclusions: that in markets with greater insurer HHI, dentists were organized into larger practices and were more likely to be non-owners, though this second result sometimes became insignificant due to 2SRI inflating the standard errors. We did not see a statistically significant relationship between insurer HHI and DMSO employment.

We also conducted of series of robustness checks. For example, we restricted the analysis to only dentists with at least 5 years of experience, used one-year lagged covariates and instruments, and replaced insurer HHI with an indicator variable representing highly concentrated dental insurance markets (HHI > 2500). These robustness checks confirmed the findings from the previously reported results and are available upon request.

## External validity check analysis

We constructed a second analytic sample as an external validity check to the ADA data used in the main analysis. The purpose of this external validity check was to confirm that the results we found for dental practices in relation to dental insurance concentration in the ADA data could be generalized to other data sources. Therefore, we constructed a county-level dataset for years 2011 through 2015 from the U.S. Census Statistics of U.S. Businesses, the AHRF, and the FAIR Health^®^ Dental Module. Our external validity check sample included 13,780 county-year observations from 2756 counties.

We measured the size of dental practices using data from the U.S. Census Statistics of U.S. Businesses (United States Census 2018). This data source provided the number of dental establishments and the number of dental establishments with five or more employees for each county and year. The dental establishment data from the U.S. Census did not include the number of dentists at a particular practice, but only provided the number of employees at that practice. Employees could include both dentists and supporting staff. Using these two measures, we constructed our dependent variable as the percentage of dental establishments that have five or more employees. From 2011 through 2015, on average, 61.6% of dental establishments had five or more employees.

We estimated a county-level fractional response model (Papke and Wooldridge 2008) where the dependent variable was the percentage of dental establishments in the county with five or more employees. Our key explanatory variable of interest was the level of dental insurer HHI. For each year, dental insurance market HHI was scaled to the county level using the Housing and Urban Development zip code-to-county crosswalk (United States Department of Housing and Urban Development 2018). The county-level dental insurance HHI was population weighted based on the number of people living in each 3-digit zip code within a county. We included a set of county controls that changed over time, including population per square mile, total dentists per square mile, dental HPSA designation, log of real median household income, and county and year fixed effects. We made a strict exogeneity assumption (Papke and Wooldridge 2008) that ruled out simultaneity and correlation between time varying omitted variables and the explanatory variables as well as lagged dependent variables. By controlling for unobserved county heterogeneity, we believed that we were accounting for unobserved factors that could influence the average partial effect of HHI.

Under a conditional normality assumption (Chamberlain 1980), APEs are reported with standard errors obtained from a panel bootstrap clustered by county with 1000 replications. For comparison, we also estimated a linear fixed-effects model to measure the association between dental insurance market concentration and dental establishment size. All regressions in this analysis were weighted by 2011 county population, although our results were robust to not weighting by population.

At the county level, we found that dental insurance market concentration had a very modest association with the size of dental establishments (Table 4). Based on estimates from fractional probit QMLE, a 1000 point increase in dental HHI was associated with a 0.35 percentage point (*p* < 0.05) increase in the percentage of dental establishments with five or more employees. The linear fixed-effects ordinary least squares (OLS) model produced nearly identical results. This shows that when using a panel data regression approach that better addresses time-invariant heterogeneity, the results we found with ADA data were externally validated with other publicly available data.

**Table 4:** County-year regressions. Dental establishment size on insurance market concentration. *Source*: 2011–2015 FAIR Health^®^ Dental Module, Area Resource File, 2011–2015 U.S. Census Statistics of U.S. Businesses

Variable	Fractional probit QMLE^a^		Linear fixed effects OLS^b^	
*Dependent variable: percentage of dental establishments with 5 or more employees*				
HHI	0.009**	0.009**	0.0036**	0.0035**
	(0.004)	(0.003)	(0.0014)	(0.0011)
Year 2012	0.008***	0.009***	0.003***	0.003***
	(0.003)	(0.003)	(0.001)	(0.001)
Year 2013	0.006*	0.007**	0.002*	0.003**
	(0.003)	(0.003)	(0.001)	(0.001)
Year 2014	0.008*	0.010***	0.003*	0.004***
	(0.004)	(0.004)	(0.002)	(0.001)
Year 2015	0.011**	0.014***	0.004**	0.005***
	(0.005)	(0.004)	(0.002)	(0.002)
Population per square mile	0.00007**		0.00003**	
	(0.00003)		(0.00001)	
Log county real median household income	0.024		0.010	
	(0.049)		(0.018)	
Dentist per square mile	− 0.012***		− 0.004***	
	(0.004)		(0.002)	
Dental HPSA	0.002		0.0009	
	(0.008)		(0.003)	
APE of HHI on dental establishment size	0.0035**	0.0035**	0.0036**	0.0035**
	(0.0015)	(0.0016)	(0.0014)	(0.0011)
Number of observations	13,780			
Number of counties	2756			

Dental insurance market concentration measured by the level of insurer HHI at the county level. Standard errors in parentheses

*HHI* Herfindahl–Hirschman index, *APE* average partial effect

***p < 0.01, **p < 0.05, *p < 0.1

^a^Panel bootstrapped standard error clustered by county, 1000 replications

^b^Robust standard error clustered By County

## Conclusion

The relationship between dental insurer concentration and the organizational structure of dental practices is not well understood. To the best of our knowledge, we are the first to show the effects of insurer concentration on the organizational structure of dental practices. Using data from the ADA and validating our results using data from the U.S. Census, we found that commercial dental insurer concentration was associated with organizational structures consistent with consolidation.

As dental insurers become more concentrated, dentists are more likely to be in larger practices and are less likely to be practice owners. There was inconclusive evidence that higher levels of dental insurance market concentration impact a dentist’s decision to affiliate with a DMSO. The magnitude of the estimated APEs of dental insurer HHI on practice size, non-ownership, and DMSO status was very modest. This may be due to significant percentage of total dental spending coming from out-of-pocket expenditures (American Dental Association 2017c). More research is needed to evaluate this possibility. Recent studies have also shown that dentists are increasingly employed rather than self-employed and are more likely to practice in large groups rather than as solo practitioners (Guay and Wall 2016; Nasseh and Vujicic 2018). Taken together, our results suggest that insurer concentration could be an important driver of these dental practice trends.

While our analysis sheds light on how the dynamics of insurance markets affect dental practice structure, the ultimate impact on consumers is still largely unknown. As commercial dental insurers continue to consolidate and gain negotiating leverage, dentists may increasingly choose to consolidate into larger practices or join large group practices. Future studies are warranted to examine whether consolidation in dental insurance and provider markets affects the price, quality and type of dental services provided to patients.
